# Transparent mediation-based access to multiple yeast data sources using an ontology driven interface

**DOI:** 10.1186/1471-2105-13-S1-S7

**Published:** 2012-01-25

**Authors:** Abdelaali Briache, Kamar Marrakchi, Amine Kerzazi, Ismael Navas-Delgado, Badr D Rossi Hassani, Khalid Lairini, José F Aldana-Montes

**Affiliations:** 1 Department of Biology, Faculty of Sciences and Techniques, University Abdelmalek Essaâdi, Tangier, BP: 416, 90000, Morocco; 2 Department of Computer Languages and Computing Science, Higher Technical School of Computer Science Engineering, University of Malaga, Malaga, 29071, Spain

## Abstract

**Background:**

*Saccharomyces cerevisiae *is recognized as a model system representing a simple eukaryote whose genome can be easily manipulated. Information solicited by scientists on its biological entities (Proteins, Genes, RNAs...) is scattered within several data sources like SGD, Yeastract, CYGD-MIPS, BioGrid, PhosphoGrid, etc. Because of the heterogeneity of these sources, querying them separately and then manually combining the returned results is a complex and time-consuming task for biologists most of whom are not bioinformatics expert. It also reduces and limits the use that can be made on the available data.

**Results:**

To provide transparent and simultaneous access to yeast sources, we have developed *YeastMed*: an XML and mediator-based system. In this paper, we present our approach in developing this system which takes advantage of SB-KOM to perform the query transformation needed and a set of Data Services to reach the integrated data sources. The system is composed of a set of modules that depend heavily on XML and Semantic Web technologies. User queries are expressed in terms of a domain ontology through a simple form-based web interface.

**Conclusions:**

*YeastMed *is the first mediation-based system specific for integrating yeast data sources. It was conceived mainly to help biologists to find simultaneously relevant data from multiple data sources. It has a biologist-friendly interface easy to use. The system is available at http://www.khaos.uma.es/yeastmed/.

## Background

The yeast *Saccharomyces cerevisiae*, known popularly as bakers' or brewers' yeast, has been used extensively in aging research. It is a unicellular organism whose DNA is packaged into chromosomes that are localized in a subcellular structure called the nucleus. Since 1990, it has emerged as an important model organism for the dissection of the biological aging process at the genetic and molecular levels. *Saccharomyces cerevisiae *was the first eukaryotic genome that was completely sequenced [1].

Nowadays, the word yeast is widely given to the species *Saccharomyces cerevisiae *because of the place it occupies in biological research. Large amounts of data related to it are genereted by Life Science and stored in multiple databases. Biologists are brought systematically to query these sources in order to analyse the results of their experiments. They usually perform the following tasks during query formulation and execution: (i) look for appropriate sources where it is possible to find helpful data and specify their location, (ii) identify the focus of each source, (iii) query each convenient source independently using its specific access method and query language, (iv) navigate through the sources to obtain complementary data, and (vi) manuallymerge the results obtained from different sources. This is a tedious and time-consuming task for biologists, most of whom are not bioinformatics experts, and reduces the advantage that can be took of the available information.

The challenges of modern bioinformatics research is not only storing data in repositories, but also processing and integrating them. Multiple solutions to biological data integration have been developed. Researchers have come up with some approaches that integrate diverse biological data sources. Two common approaches have being used to interoperate biological databases: data warehousing (or materialised) approach [2] and federated/mediator-based (or virtual) approach [3].

The data warehousing approach is adopted by numerous biological integration systems like GUS [4], Atlas [5], BioSQL [6], BioMart [7], BioWarehouse [8], and chado [9]. This approach uses a data warehouse repository that provides a single access point to a collection of data, obtained from a set of distributed, heterogeneous sources. Data from the remote heterogeneous databases are copied on a local server and the user will use a unique interface within the system to allow multi-database queries to be issued to this single interface. Data warehousing requires the use of Extraction, Transformation and Load (ETL) [10] tools to load data, and map it to a materialised global schema. In fact, warehousing requires that all the data loaded from the sources be converted through data mapping to standard unique format before it is physically stored locally. Relying less on the network to access the data clearly helps to eliminate various problems such as network bottlenecks, low response times, and the occasional unavailability of sources. Furthermore, using materialised warehouses allows for an improved efficiency of query optimisation as it can be performed locally [11,12]. Another benefit in the data warehouse integration approach is that it allows the system to filter, validate, modify, and annotate the data obtained from the heterogenous sources and this has been noted as a very attractive property for bioinformatics. This approach however has an important and costly drawback in terms of reliability of results and overall system maintenance caused by the possibility of returning outdated results. Warehouse integration must indeed regularly check all the underlying sources for new or updated data and then reflect those modifications on the local copy of the data [12].

Virtual integration (mainly mediator-based systems) concentrates on query rewriting: It rewrites the user query, into queries that are understood by the integrated sources. The mediator uses the relationships between sources and a global schema to translate queries on the mediator schema to the data source schemata. The two main approaches for establishing the mappings between each source schema and the integration schema are global-as-view (GAV) and local-as-view (LAV) [11,13]. In the GAV approach the mediator relations are directly written in terms of the source relations. The GAV approach greatly facilitates query reformulation as it simply becomes a view unfolding process. In LAV approach every source relation is defined over the relations and the schema of the mediator. It is therefore up to the individual sources to provide a description of their schema in terms of the global schema, making it very simple to add or remove sources but also complicating the query reformultaion and processing role of mediator.

The mediator-based approach has several strengths compared to data warehouse. It does not have the updating problem as the query goes directly to the original source. Mediators can be seen as a cheaper and more effective approach since they use schema or view integration, rather than having to have huge storage capacity to store copied data from all the involved data sources.

This paper presents a mediator-based system called *YeastMed *[14] that aims to provide transparent access to disparate biological databases of yeast. It provides a unique interface between the user who submits a query, and a set of five data sources accessible via web protocols. *YeastMed *relies on SB-KOM [15] to perform the query transformation needed to reach the integrated data sources. These sources are: *SGD *[16], *Yeastract *[17], *CYGD-MIPS *[18], *BioGrid *[19] and *PhosphoGrid *[20]. They provide complementary data on biological entities (cellular interaction, metabolic pathways, transcription factors, annotation data...). With *YeastMed*, we aim to help biologists to get relevant data to understand and explain the biological processes of interest by using an integrative system.

This paper is organised as follows: an overview on some biological data integration systems is given in the next section. Then, a general overview of the system and the resources used in *YeastMed *are given before to describe the integration process components along with some explanatory schemas. A detailed use case is then sketched describing how *YeastMed *proceeds when a user query is submitted. At the end, we discuss some advantages and limitations of the current version of *YeastMed *before to conclude the paper.

### Related work

Works specific to the integration of yeast data sources are not abundant. However a variety of data integration systems especially tailored to cater for bioinformatics applications have been developed. These systems can broadly be classified as: data warehouses, federated/mediator-based systems and XML-based systems.

#### Data warehouse systems

Several attempts have been made to create integrated environments for storing and analysing biological data. For the sake of brevity, we sketch here two of them due to their relation to yeast.

Cell Cycle Database [21] is an integrated data warehouse for systems biology modelling and cell cycle analysis based on yeast and mammalian organisms. The system integrates information about genes and proteins involved in the cell cycle process. It stores complete models of interaction networks and allows the mathematical simulation over time of the quantitative behaviour of each component. The database integration system consists of a series of programs used to retrieve the data from several different external databases, transform and load them into the warehouse data model.

YeastHub [22] is a prototype application in which a data warehouse has been constructed in order to store and query different types of yeast genome data provided by different resources in different formats including the tabular and RDF formats. Once the data are loaded into the data warehouse, RDF-based queries can be formulated to retrieve and query the data in an integrated fashion. YeastHub is implemented using Sesame 1.1 [23]. The tabular-to-RDF conversion is written using Java.

These two systems present some limitations: they store the extracted data locally in a data warehouse or database which render the updating process a tedious task. YeastHub presents another problem where Sesame does not have a way to identify the source of the triples (statements) once they are loaded into the repository. In contrast, *YeastMed *accesses and interrogates data in its original data sources, and provides the user with the possibility to choose which data source entry to return. If the user doesn't make a choice, the system explicitly gives the provenance of the result entries.

#### Federated and mediator-based systems

Other alternative solutions have been proposed in biological data integration adopting a virtual approach. Among them we can cite:

Kleisli [4] is as a mediator system encompassing a nested relational data model, a high-level query language, and a powerful query optimiser. It runs on top of a large number of light-weight wrappers for accessing various data sources. The Kleisli system is highly extensible. It can be used to support several high-level query languages by replacing its high-level query language module. Kleisli supports the Collection Programming Language (CPL) [24] and a nested relational version of SQL. However Kleisli does not use any global schema or ontology over which a user can formulate queries. A query attribute is bound to a matched attribute in single source, so there is no integration across different sources.

DiscoveryLink [25] is a wrapper-oriented bioinformatics integration system built on the Garlic project technology [26]. It serves as a middleware between the applications and a set of wrappers. Applications connect to DiscoveryLink and submit an SQL query on its global schema. The wrappers provide source-specific information about query capabilities that help the optimiser to determine which parts of a query can be submitted to each source. The query optimiser considers the speed of various sources, their network connections, and the size of their data to predicate the costs of different plans. DiscoveryLink, however, cannot deal with complex source data such as nested data. Most biological data, unfortunately, are highly nested. Therefore, there is a significant amount of mismatch between most data sources and DiscoveryLink. Furthermore, it is hard to add new data sources or analysis tools to DiscoveryLink. In addition, DiscoveryLink requires SQL as its query language, which is not easy for biologists to write.

TAMBIS [27] is a mediator-based and ontology-driven integration system, it has three layers: the conceptual model, the mapping model and the physical model. In TAMBIS, the formulation of queries is done through a graphical interface where user needs to browse through the different concepts defined in the global schema and select the suitable ones for particular query. As the first step, the system expresses the graphical query in GRAIL [28]. Then, the query is translated into a Query Internal Form (QIF), which is in turn translated into a source-dependent query execution plan in CPL [24]. The global ontology is a unified conceptual-level representation of its registered component resources. It provides a global schema as well as an abstract framework for relating, reconciling, and coordinating the concepts in the sources. The mapping model converts a query phrased in terms of the conceptual layer into executable plans in terms of each source. The physical model submits the executable plans to different sources and retrieves the results. Although TAMBIS is more of an upper level solution than other systems, but its graphical interface is very complicated and requires that a user understands the query language. BioMediator [29] is a federated data integration system based on XML. It uses a mediated schema which allows for more flexible data modelling. The central component of BioMediator system is its source knowledge base, which consists of descriptions of the various data sources, mappings from the source to the mediated schema, and the mediated schema itself. The system include also wrappers that conduct syntactic translations by translating the returned data results into an XML document, a metawrapper that conducts semantic translations by mapping the returned XML document onto the mediated schema, and a query processor that queries (using XQuery Language) against the mediated schema. BioMediator is thus dedicated to users who know the XQuery language and is not willing to be used by external research groups.

Compared to these systems, *YeastMed *is the first data integration system which adopts the mediation approach to integrate yeast-specific data sources. It has a domain ontology which plays the role of the global schema and supports the user queries. Unlike the systems cited above, *YeastMed *has an easy-to-use ontology driven interface where users express their requests in simple natural language. Users do not need to know a specific query language to use it. In addition, due to its modular design, *YeastMed *furnishes the possibility to add easily new data sources or analysis tools.

#### XML-based systems

Despite the possibility to use standard approaches for data integration [30], specific approaches based on the employment of XML in Bioinformatics have been proposed: 

Automed [31] is a heterogeneous data transformation and integration system which offers the capability of handling virtual, materialised and hybrid data transformation/integration across multiple data model. AutoMed uses XML DataSource Schema (XMLDSS) as a common representation language and schema type supporting the annotations for each source by suitable ontologies. An XMLDSS schema can be automatically extracted from an XML document or automatically derived from an accompanying DTD/XML schema if one is available.

The system approach is based on: (i) XML as a common representation format; (ii) XMLDSS as the schema type for the XML documents input to and output by services; (iii) Correspondence to available ontologies; i.e. the services inputs/outputs are annotated with correspondences between the XMLDSS schema and some existing ontologies; and (iv) AutoMed toolkit to automatically transform the XMLDSS schema to output of a given service to the XMLDSS schema of the input of another service. 

SWAMI [32] defines a rich middleware architecture that integrate different databases, formats and computational resources. Its architecture design includes a *Presentation layer *that receives user requests, passes them to the *Core workbench Application*, and returns application results to user by the same route. The Core Application consists of four major components: *The user module *which receives data and instructions from the Presentation Layer. *The Broker module *which interacts with the others modules via APIs and serves as coordinator using a registry service that maintains information about all available services and databases. Then *The Tool *and *Data modules*, which are conceptually identical, abstract respectively applications and databases, and perform their functions by orchestrating a series of services. XML is used for the declarative specification of services.

## Methods

*YeastMed *is a mediator-based system that consists of several components contributing to the data integration process in different ways. In this section we talk in detail about the process for creating the system by giving descriptions of its components and the role of each of them.

### YeastMed overview

**Figure 1:**
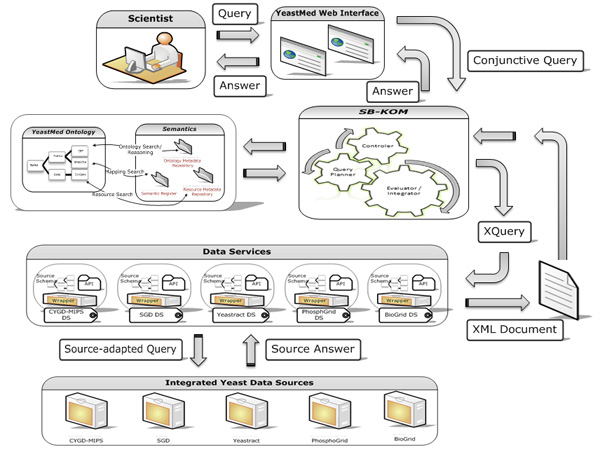
**General architecture of YeastMed system**. It shows how the different components of YeastMed System are structured and interact between them.

The general architecture of the *YeastMed *system is shown in (Figure 1). It consists of a set of components that have been implemented independently and play different roles. The access point to the system is a web interface that furnishes two search forms:

▪ A quick search form where scientists can quickly submit their requests based on some keywords (Gene or Protein names, GO terms or any other words that can appear in the search fields of the interrogated data sources). This type of search does not make use of the mediator. It exploits the *YeastMed web *services to look for information in the integrated data sources.

▪ An ontology-driven search form which allows biologists to express their requests in terms of the *YeastMed Ontology*. These terms are presented in natural language to ease the query formulation process for biologists most of whom are not familiar with knowledge representation and query languages.

*YeastMed *relies on SB-KOM [15] to perform query transformation at execution time. Once the user submits a request from the web interface, *YeastMed *generates a conjunctive query. SB-KOM decomposes this query into suitable sub-queries to individual sources based on a set of mapping rules. These sub-queries are expressed in XQuery, because the sources are accessed through web services using this query language.

*YeastMed *have a set of web services (Data Services for us): one for each integrated source. These components receive XQueries from SB-KOM and return XML documents. The role of the web services is to allow *YeastMed *to use wrapper functionalities to find and extract solicited information from data sources through their web pages or FTP mechanisms. Answers, materialised by XML documents, to XQueries are sent to the mediator which combines them into a *YeastMed *ontology instance expressed in RDF. The final result is provided for the user in HTML format. Data sources are also an important component in the *YeastMed *architecture because they are the providers of the biological information.

### Integrated data sources

In its current version, *YeastMed *integrates five Yeast databases. They have been selected for having the most appropriate properties for studying *Saccharomyces cerevisiae*, because they provide complementary data concerning genome, proteome, metabolome and reactome. These sources are:

▪ SGD Database [16]: It contains the sequences of yeast genes and proteins, descriptions and classifications of their biological roles, molecular functions, subcellular localisations, links to literature information and tools for analysis and comparison of sequences.

▪ YEASTRACT Database [17]: It is a repository of regulatory associations between transcription factors and target genes, based on experimental evidence which was spread throughout bibliographic references. Each regulation has been annotated manually, after examination of the relevant references. The database also contains the description of specific DNA binding sites for a sub-group of transcription factors.

▪ MIPS-CYGD [18]: aims in general to present information on the molecular structure and functional network of *Saccharomyces cerevisiae*. In addition, the data of various projects on related yeasts are also used for comparative analysis.

▪ BioGRID [19]: It is an online interaction repository with data compiled through comprehensive curation efforts. All interaction data are freely provided through the search index and available via download in a wide variety of standardised formats.

▪ PhosphoGRID [20]: records the positions of specific phosphorylated residues on gene products. Where available for specific sites, PhosphoGRID has also noted the relevant protein kinases and/or phosphatases, the specific condition(s) under which phosphorylation occurs, and the effect(s) that phosphorylation has on protein function.

### YeastMed user interface

**Figure 2:**
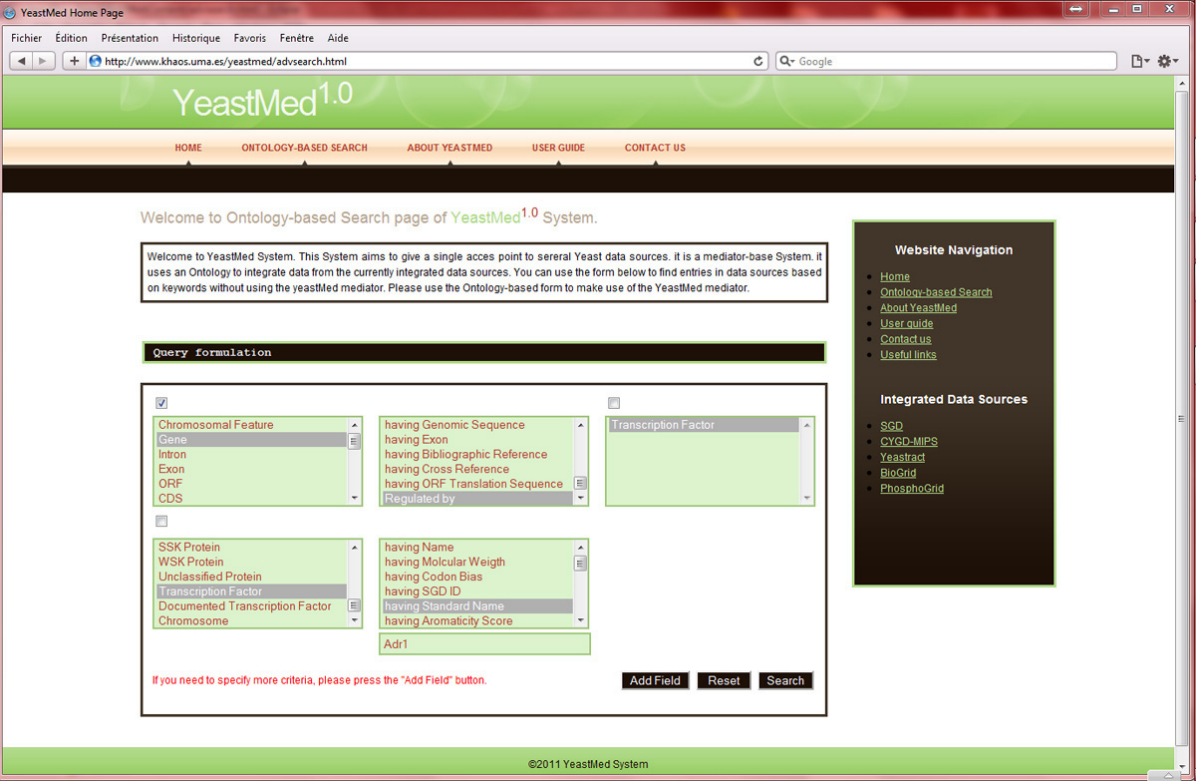
**The ontology-based search interface of YeastMed**. It shows how is captured the example sketched in this section.

The fact that biologists are familiar with HTML forms when interrogating biological databases, and in order to make *YeastMed *easy to use, we have adopted the same strategy that most biological databases are using to receive queries: A simple HTML-form-based interface has been developed permitting the queries to be expressed in natural language. It is an ontology driven interface. Users formulate their queries by selecting items from the form fields. These items have their equivalents in the *YeastMed *ontology (concepts and properties) and are written in natural language. For example the concept *BibRef *in the ontology is translated in the form fields as *Bibliographic Reference *and the datatype property *hasProductDesc *as *having Product Description*. We are convinced that it is very easy for users to express in natural language their requests by using implicitly triplets composed of, those designed in the terminology of ontologies by, domain, property and range. For example, the user interested in the set of genes regulated by the transcription factor having the standard name Adr1, can express it using the two assertions: "Gene regulated by Transcription Factor" and "Transcription Factor has standard name Adr1". In this context, we have designed the YeastMed interface to capture these kinds of expressions. The query form proposes three fields per line. Each line represents the triplet formed by: domain, property and range. Range can be either a concept to select from the third field in a line or a literal value to introduce in a field that appears at the bottom of the second field if a datatype property has been selected in it. The example above can be captured in *YeastMed *interface using two field lines as follows: in the first line "*Gene*" "*regulated by*" "*Transcription Factor*" and in the second line "*Transcription Factor*" "*having Standard Name*" "*Adr1*" (Figure 2). When submitted, the system makes use of the equivalents of these in the ontology and creates the conjunctive query: Ans(G):= Gene(G), regulatedBy(G, TF), TranscriptionFactor(TF), hasStandardName(TF, "Adr1") before to send it to the mediator component.

The *YeastMed *web site also gives the possibility for users to use a quick search form to interrogate the five integrated databases without using the mediator. Users simply enter their keywords in an input field, select the databases to be looked up and the system takes advantage of the *YeastMed *Data Services to access and extract data from the underlying sources.

### Data integration in YeastMed

*YeastMed *has a set of modules that depend heavily on XML and semantic web technologies to integrate syntactically and semantically biological data. In what follows, we give detailed information on these components.

#### Source schemas

**Figure 3:**
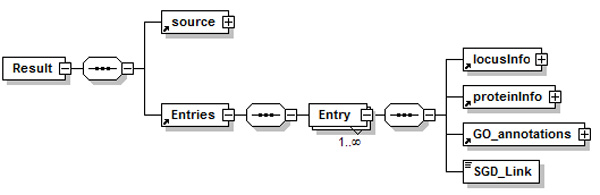
**A fragment of the yeastract schema**. It is used in YeastMed as a model describing data and their organisation in data source and defines a structure under which results will be returned by the Yeastract web service.

The knowledge modelling of the application domain of *YeastMed *constitutes the corner stone for an efficient integration. To that end, a detailed study of the sources has been carried out with the goal of establishing a standard terminology to describe the data. Each data source has been modelled by an exported XML Schema (Figure 3). An exported schema refers to translated source schema in the *YeastMed *Ontology. These schemas are considered as models describing data and their organisation in data sources and define a structure under which results will be returned by Data Services.

#### Data services

*YeastMed *uses a set of web services (called in our case Data Services) to access data sources. We have developed one Data Service for each integrated yeast source. These components hide technical and data model details of the data source from the mediator. They receive XQueries from SB-KOM and return XML documents in addition to other metadata. The role of *YeastMed Data *Services is twofold:

▪ Allowing *YeastMed *to use the wrapper functionalities to find and extract solicited information from data sources using HTML protocols or FTP mechanisms. This means providing the ability to solve XQueries and return answers in XML format.

▪ Exporting semantic information about data schemas and data provenance. This allows mainly *YeastMed *to keep track of the returned information when combining them and also which source is being interrogated.

**Figure 4:**
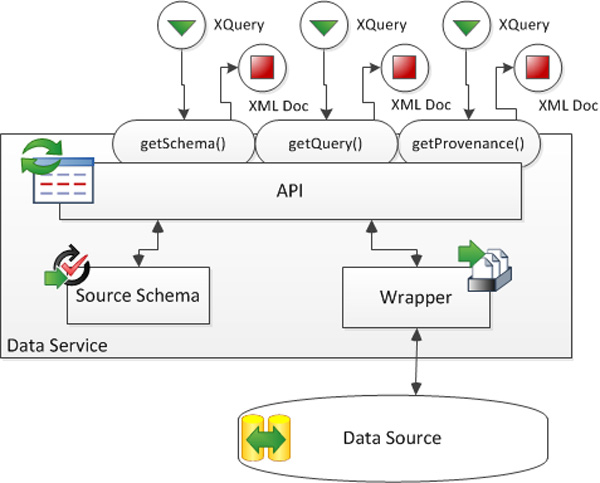
**Architecture of the web services in YeastMed system**. Services receive Xqueries through the different methods of the API and transmit them to the wrapper. The output is an XML document.

It is common knowledge that a wrapper is an interface for a data source that translates data into the common data model used by mediators [33]. Because the goal of *YeastMed *is to integrate databases accessible via Web protocols, it is completely normal that a wrapper is considered as the most important component of the architecture of *YeastMed *Data Services. It is an interface that receives XQueries generated by SB-KOM, accesses a specific data source, extracts data and translates them into the common data model used by SB-KOM, i.e. XML (Figure 4).

In addition to the wrapper's query service, the web services encapsulate an Application Programming Interface (API). It is the access point for SB-KOM to the functionality of the web service. This API publishes three methods: *getQuery(Q) *that passes to the wrapper the XQuery *Q *and returns its answer in an XML format. The XML structure of this answer must satisfy the constraints of the source schema. The other two methods, *getSchema() *and *getProvenance()*, provide access to the metadata that the web service stores. The former returns the XML data schema and the latter provides information on the underlying data source. In order to use these methods correctly, SB-KOM finds all the necessary information about them in a WSDL (Web Service Description Language) document.

The Data Services have been implemented in Java. They receive XQueries from SB-KOM via the *getQuery() *method of the API which passes it to the wrapper. This is materialised by a set of java classes that define several methods. The incoming XQuery is analysed to identify precisely what information is solicited from the underlying data source. The wrapper then generates a source-adapted query following the query capabilities of the source already specified for each Data Source. Then it establishes a connection to the data source via HTML or FTP protocols. In the case of HTML protocol, the data source is interrogated through its web interface using its query engine. The answer is one or several web pages which are parsed on the fly to extract the solicited data. In the case of FTP protocol, the data source is interrogated through its available flat files which are also parsed on the fly. A set of methods are defined to extract data from source answers and organize them as an instance of the XML source schema before to send it to the SB-KOM in the form of an XML document.

*YeastMed *is able to reflect data provenance by calling the method *getProvenance() *which returns information about a source or through the XML document returned by Data Services: it contains by default a description of the interrogated data source. Thus, instances with the integrated data can be annotated with the data provenance of each piece of information. In this way, the user interface could show users the provenance of each part of the results.

#### YeastMed ontology

As mentioned before, the goal of the *YeastMed *System is to help scientists to get information from multiple Yeast data sources by providing a single access point. To that end, we have equipped *YeastMed *with a domain ontology. The primary purpose of this Ontology is to support the user queries. Queries are phrased in terms of the ontology and *YeastMed *converts these to XQuery requests to the appropriate sources via Data Services. The *YeastMed *ontology has been constructed from scratch by reconciliating the different data source schemas into a single, coherent ontology.

**Figure 5:**
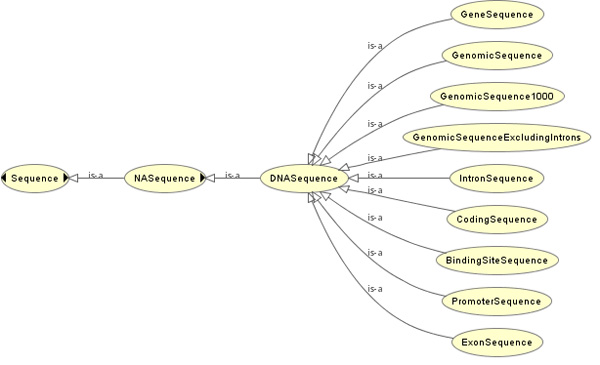
**A fragment of the YeastMed ontology**. It shows the semantic encapsulation of the concepts related to DNA sequences in *YeastMed*. For example the concept *GeneSequence *which represents the set of gene sequences is a child of the concept *DNASequence *which represents all type of DNA sequences. *DNASequence *is in turn a child of *NASequence *which represents the set of nucleic acid sequences.

The *YeastMed *ontology [34] ensures semantic encapsulation of data sources by defining a concepts hierarchy. This is a classification of all the biological entities manipulated by the system. It represents a knowledge model that captures biological and bioinformatics knowledge in a simple hierarchical conceptual framework constrained by parent-child relationships (Figure 5): A child is a subset of a parent's elements; each child inherits all of its parent's properties but has more specialised properties of its own. Overall, the ontology concepts can be classified into two categories: the purely biological concepts category and the source-related concepts category.

▪ The purely biological concepts category, which is a union of all the classes modelling biological entities found in the integrated data sources. As an example of this category, we cite *ChromosomalFeature *concept. It is the superclass of 38 classes representing different types of chromosomal features (genes, CDS, intron, repeat regions, etc).

▪ The source related concepts category is represented by concepts referring to sources. For example the concept *Source* represents the five integrated data sources and the concept *Entry* refers to entries in data sources. Adding this category to the ontology has as the objective to permit scientists, when using *YeastMed*, to express their preferences on data sources. So, giving the possibility to determine which source entry they want *YeastMed *to return if a result is found rather than the system making its own choice.

To convey additional semantic information about the concepts, the ontology defines two types of properties. The first one is defined by a set of object properties that model the relationships that can hold between two individuals belonging to one or two different classes of the ontology. The second type concerns data properties: these are relationships linking an individual to a literal data.

**Figure 6:**
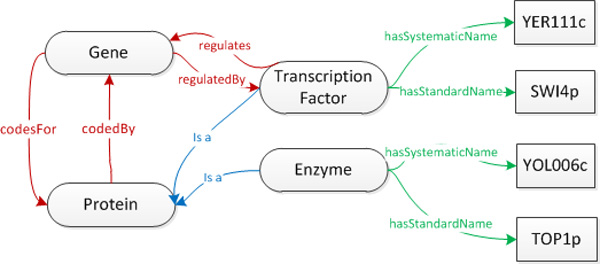
**A schematic representation of the example sketched in this section**. It shows four biological concepts (ellipses) linked by four object properties (red arrows) and two parent-child relationships (blue arrows), and two datatype properties (green arrows) linking two concepts to values of type String (rectangles).

To further illustrate the role of properties in conveying semantics to the *YeastMed *ontology, we detail a real-world example (Figure 6). *SWI4 *[35], having the systematic name *YER111c*, is a gene coding for a DNA binding component of the SBF complex (Swi4-Swi6), a transcriptional activator that in concert with MBF (Mbp1-Swi6) regulates late G1-specific transcription of targets including cyclins and genes required for DNA synthesis and repair, an example is *Topoisomease I *[36] (which have the standard name *TOP1)*. From this we can make the following assertions:

▪ SWI4 and TOP1 are two Genes having the systematic names YER111c and YOL006c;

▪ SWI4 and TOP1 code respectively for a Transcription Factor and an Enzyme;

▪ SWI4 regulates the transcription activity of TOP1;

▪ Both SWI4 and TOP1 code for proteins (having respectively the same standard names as their Genes).

These assertions let one define:

▪ Four concepts: *Gene, Protein, TranscriptionFactor*, and *Enzyme*;

▪ Four object properties: *codesFor *and its inverse property *codedBy *linking *Gene *to *Protein*, in addition to the property *regulates *and its inverse *regulatedBy *linking *TranscriptionFactor *to *Gene*;

▪ Two datatype properties: *hasSystematicName *and *hasStandardName *linking *TranscriptionFactor *and *Enzyme *to literal values of type String (SWI4 and TOP1 for the former and YER111c and YOL006c for the latter);

▪ *Enzyme *and *TranscriptionFactor *as child concepts of *Protein*.

In *YeastMed*, we have chosen OWL [37] as a standard Ontology language to represent the ontology. OWL is, like RDF [38], taking advantage of the syntactic universality of XML. Based on the RDF/XML syntax, OWL provides a way to write web ontologies. It is different from the couple RDF/RDFS in the sense that is just a language of ontologies: If RDF and RDFS bring the user the ability to describe classes (with constructors) and properties, OWL incorporates, in addition, comparison tools for properties and classes: identity, equivalence, contrary, cardinality, symmetry, transitivity, disjunction, etc. Thus, OWL offers for machines a greater capacity of interpretation of the web content than RDF and RDFS [39], with a wider vocabulary and a real formal semantics. To be more precise, we have contented ourselves with using OWL-Lite (which is a sub-language of OWL) because we have envisaged from the beginning to equip *YeastMed *with a simple domain ontology showing a simple concepts hierarchy and simple constraints.

#### Mappings

Having a domain ontology facilitates the formulation of queries to the system. The users simply pose queries in terms of the ontology rather than directly in terms of the Source Schemas. Although this is very practical and effective in terms of the system transparency to the user, it brings the problem of mapping the query in the mediated schema to one or more queries in the schemas of the data sources. In *YeastMed*, this problem is solved using the functionality of SB-KOM. So in addition to modelling the ontology and the sources, we needed to establish associations between the concepts in the ontology and the appropriate elements representing the information in the sources. These associations are materialised in *YeastMed by *the mapping rules.

SB-KOM is designed to decompose queries based on GAV approach-based mappings. That means each concept (also property in our case) in the ontology is a view defined in terms of the source schemas' elements. This view specifies how to obtain instances of the mediated schema elements from sources. In this context, the mapping rules we have used are defined as pairs (P,Q). P is one or a couple of path expressions on a source schema expressed in XPath, and *Q *a conjunctive query expressed in terms of the Ontology terms. Three kinds of mappings have been defined:

▪ Class Mapping: it maps ontology classes to source schemas. It has the following form:

XPath-Element-Location, Ontology-Class-Name, correspondence-index

Where *XPath-Element-Location *is the location of an element in the source schema, expressed in XPath; *Ontology-Class-Name *is the name of the corresponding class in the Ontology and *correspondence-index *is an integer value that informs on the correctness of the mapping instance. In *YeastMed*, this index is always 100 since all the mappings are done manually and not automatically. An example which maps the *Protein *class to the SGD schema is as follows:

Result/Entries/Entry/Protein, Protein,100

▪ Datatype Property Mapping: it maps ontology datatype properties to source schemas. It has the following form:

XPath-Domain-Location; XPath-value-Location, Ontology-Domain-Name; Property-Name, correspondence-index

*XPath-Domain-Location *is the Path to the element in the source schema which is mapped to the domain of the datatype property; *XPath-value-Location *is the Path to the element where the property takes the value of its range and *Ontology-Domain-Name *and *Property-Name *are respectively the domain and the name of the property. The following example concerns the datatype property *hasName:*

Result/Entries/Entry/Protein; Result/Entries/Entry/Protein/SysName, TranscriptionFactor;has Name,100 

 ▪ Object Property Mapping: it maps ontology object properties to source schemas. It has the following form:

XPath-Domain-Location; XPath-Range-Location, Ontology-Domain-Name; Ontology-Range-Name; Property-Name, correspondence-index

*XPath-Range-Location *is the Path to the element in the source schema which is mapped to the range of the object property. *Ontology-Range-Name *is the range name of the property. The following example shows how the object property *hasBibRef is *mapped to the source schema:

Result/Entries/Entry/Protein;Result/Entries/Entry/Literature, Protein;BibRef;hasBibRef,100

#### SB-KOM

*YeastMed *relies on SB-KOM [15], to perform query transformations at execution time. KOMF is a generic infrastructure to register and manage ontologies, their relationships and also information relating to the resources. This infrastructure is based on a resource directory, called Semantic Directory [40], with information about web resource semantics. KOMF has been successfully instantiated in the context of molecular biology for integrating biological data sources [41-43]. SB-KOM mediator is composed of three main components: the Controller, the Query planner and the Evaluator/Integrator.

The Controller component receives requests coming from the YeastMed web interface and evaluates them to obtain a result for the requests. The controller creates different threads for different user requests, and assumes the role of the middleware between the mediator components. Queries are expressed as conjunctive predicates [44], with three main types of predicate: classes in terms of YeastMed ontology which is registered in the Semantic Directory, datatype properties that link individuals to data values, and object properties that link individuals to individuals. The results of these queries are instances of the YeastMed ontology which the query was expressed in.

The Query planner component is by far one of the most fundamental pillars in elaborating one or several query plans to solve the query from different data sources. Plans generated by this component specify the data sources from which the information can be retrieved and in which order they must be accessed. The evaluation of these queries depends on the query plans themselves.

According to the query (a conjunctive query), there will be different types of mapping in the Semantic Directory. Classes will be connected to the XPath of one or several XML Schema resource elements. On the other hand, datatype properties will be connected to those two expressions: the first one corresponds to the class and the second to the property. The object properties will be related to the active XPath classes in the property.

The Query Planner runs following a simple algorithm that receives as entry a conjunctive query expressed in terms of the *YeastMed *ontology (conjunction of concepts and properties) and returns a set of possible plan trees. The algorithm steps are enumerated below (for a use case see the following section):

1. Get all the query predicates (concepts and properties) and distribute them in two groups based on the number of the arguments: Ɠ_*1 *_will contain predicates having one argument (concepts) and Ɠ_*2 *_will contain predicates having two arguments (properties).

2. Construct a set Ƈ_*s *_of combinations between the two groups based on common arguments, add all the elements of Ɠ_*1 *_and Ɠ_*2 *_to it and eliminate the repeated ones.

3. Eliminate from Ƈ_*s *_the elements that do not have a representation in the mapping rules registered in the Semantic Directory.

4. For each instantiated variable in the predicate arguments, elaborate a plan tree:

a. The instantiated variable will construct a root node.

b. The elements that contain a predicate specifying a value for the instantiated variable and the elements that contain only the instantiated variable (without other variables) will be passed to the current node and eliminated from Ƈ_*s*_.

c. The elements that contain, in addition to the instantiated variable, another variable will constitute the edges leaving the current node to new nodes and eliminated from Ƈ_*s*_. The newly created nodes will be represented by the other variables which will be the instantiated variables.

d. if there are still more elements in Ƈ_*s *_and for each new instantiated variable we continue from the step 4.b.

The Elvaluator/Integrator is the third component of SB-KOM mediator. It analyses the query plan (QP), and performs the corresponding calls to the Data Services involved in the sub-queries (SQ1, ..., SQn) of the query plan. To answer YeastMed query, this component first executes the Data Services in the order specified by the query plan. Then, it obtains the instances from the Data Service results. These instances are not interconnected because they have been produced by different Data Services. In order to retrieve a set of interrelated instances we need to establish relationships between them. This can be achieved by the object properties defined in the ontology that are used as relationships between services in the query plan. Finally, these interrelated instances are filtered in order to eliminate the information not required.

### Use case

In this section, we show how a user query is solved by *YeastMed*, and how its different components take part in this process. Let us take the case of a biologist who is using *YeastMed *to find information about two kinds of proteins. The first one is represented by DNA Topoisomerase III, and the second one is indicated by some transcription factors regulating the expression of the first kind. The biologist is interested in the phosphorylation sites that are found in the sequences of the transcription factors of DNA Topoisomerase III, especially the one (or ones if they exist) whose gene is located on the Chromosome 16. In addition, the biologist also aims to get all the literature on DNA Topoisomerase III. As stated previously, *YeastMed *provides a web interface that allows biologists to express this kind of requests in terms of the ontology. The user can formulate its request in the *YeastMed *interface by selecting fields' items as follows:

"*Protein*", "*having Description*", "*DNA Topoisomerase III*";

"*Protein*", "*having Bibliographic Reference*", "*Bibliographic Reference*";

"*Protein*", "*Regulated By*", "*Transcription Factor*";

"*Transcription Factor*", "*Belongs To*", "*Chromosome*";

"*Chromosome*", "*having Name*", "*16*";

"*Transcription Factor*", "*having Phosphorylation Site*", "*Phosphorylation Site*".

To specify to the system what to return, the user should add checkmarks by clicking on the boxes above the fields where "*Bibliographic Reference*" and "*Phosphorylation Site*" were chosen before to submit its query.

**Figure 7:**
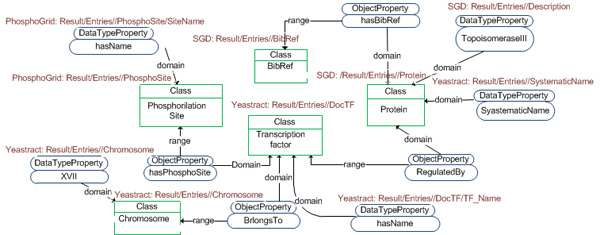
**The fragment of the ontology invoked to formulate the query example**. Classes are shown in green and Properties in blue. The mappings between the ontology and the source schemas are present above the ontology element (in red).

The fragment of semantics that is implied directly in the formulating process of that query is shown in (Figure 7). From this fragment, a conjunctive query is generated automatically:

Ans(BR,Ph):= Protein(P),hasDescription(P,"DNA Topoisomerase III"), BibRef(BR),hasBibRef(P,BR), hasSystematicName(P,SN), regulatedBy(P,TF),hasName(TF,Nt),TranscriptionFactor(TF),Chromosome(C),hasName(C,"16"), BelongsTo(TF,C),PhosphoSite(Ph), hasPhosphoSite(TF,Ph);"

This conjunctive query includes as predicates five ontology classes (*Protein*, *BibRef*, *TranscriptionFactor*, *Chromosome *and *PhosphoSite*), three datatype properties (*hasDescription*, *hasSystematicName *and *hasName*) and four object properties (*hasBibRef*, *regulatedBy*, *belongsTo *and *hasPhosphoSite*). This query will return instances of *PhosphoSite *and *BibRef *that satisfy its constraints.

As a subsequent step, the conjunctive query will be sent to SB-KOM, received by the controller which will pass it to the Query Planner. This component has an algorithm that, based on the query predicates and the mappings of the semantic directories, will generate a set of sub-queries and also a plan to execute them. The predicates of the conjunctive query are divided into two sets: a set that contains predicates with a single argument and another that contains predicates with more than one argument. The predicates from the two sets which have common arguments are then grouped together into groups represented by the combination of two or more predicates. The groups that are not represented in the Semantic Directory mappings are discarded. The remainder is added to the first set allowing a group to be present only once. (Table 1) lists all resulting groups.

**Table 1:** The groups used to form the plan tree.

Group	Query	Mapping source
G1	Protein(P), hasBibRef(P,BR)	SGD
G2	Protein(P),hasDescription(P,"DNA Topoisomerase III")	SGD
G3	Protein(P), hasSystematicName(P, SN)	Yeastract
G4	Protein(P), RegulatedBy(P, TF)	Yeastract
G5	TranscriptionFactor(TF), hasName(TF, Nt)	Yeastract
G6	TranscriptionFactor(TF), belongsTo(TF,C)	Yeastract
G7	TranscriptionFactor(TF), hasPhosphorylationSite(TF, Ph)	PhosphoGrid
G8	Chromosom(C), hasName(C,"16")	Yeastract
G9	regulatedBy(P,TF)	Yeastract
G10	hasBibRef(P,BR)	SGD
G11	belongsTo(TF,C)	Yeastract
G12	hasPhosphoSite(TF,Ph)	PhosphoGrid
G13	Protein(P)	SGD; Yeastract; PhosphoGrid
G14	TranscriptionFactor(TF)	Yeastract; PhosphoGrid
G15	BibRef(BR)	SGD
G16	Chromosome(C)	Yeastract
G17	PhosphoSite(Ph)	PhosphoGrid

For each group the mapping source is given.

**Figure 8:**
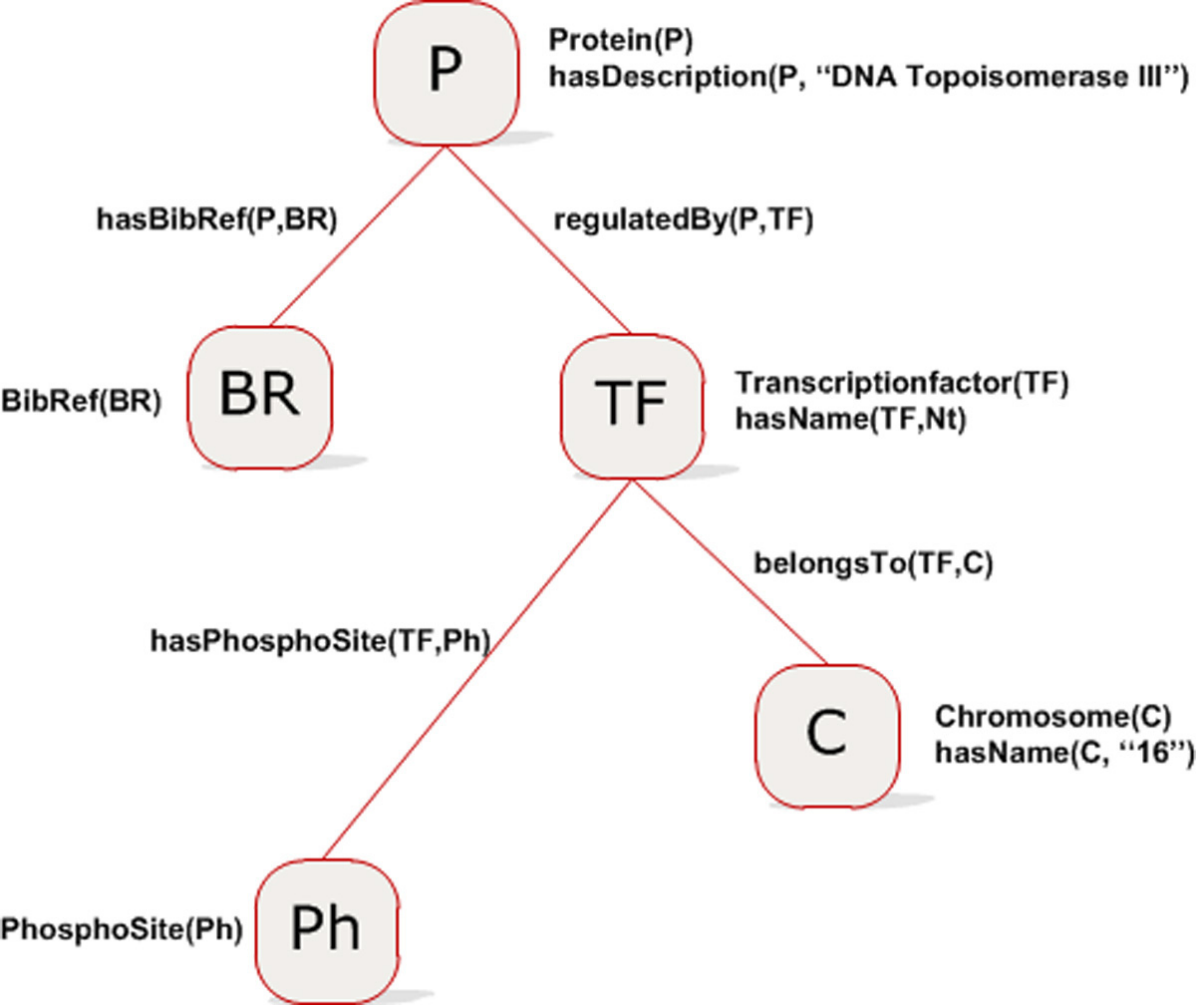
**The plan tree generated from the conjunctive query**. The plan tree is a binary tree where nodes are represented by variables of the predicate arguments in the conjunctive query and the edges are predicates containing the two variables of the nodes they are linking.

**Figure 9:**
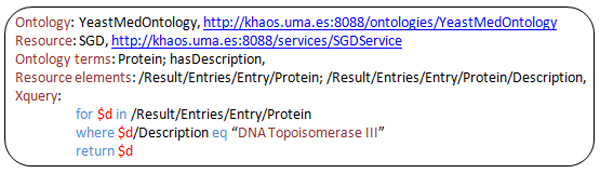
**The information presented by the node P in the plan tree**. The node P contains the location of the YeastMed ontology and the web service to call, in addition to the mapping resources and the xquery to send to the web service.

From this set, the planner will try to construct potential trees of the execution order. It selects groups with variables instantiated in order to set a root for a tree. The order of the plan execution depends on the instantiated variables: the group containing an instantiated variable is executed first, then the groups that are related to those variables, and so on until all the groups are executed. In our case, G2 and G8 are selected. G8 cannot serve as a root, because there is no other group that depends on its instantiated variable which keeps the other groups without execution. This is not the case for G2 which serves as a root for the tree shown in (Figure 8). It is the first to be executed. This returns the protein that has as description "DNA Topoisomerase III". Then G9 and G10 are executed in parallel because they depend on the instantiated variable of G2. From these simultaneous executions, the algorithm will determine all the objects that are related to *Protein *by means of the relationships *regulatedBy *and *hasBibRef*. Once those objects are obtained, it will check whether they satisfy G14 and G15: that means checking if the objects obtained from G9 and G10 are respectively of the type *TranscriptionFactor *and *BibRef*. Based on the result of G9, groups G11 and G12 are executed but not simultaneously. SB-KOM has a plan optimisation module that might change the order of the initial plan execution as is the case here: Since G8 has a variable instantiated (value "16") and is related to G14 via G11, this one is executed before G12, and the result is used by the group to be executed. The arcs of the planning trees generated by the planer represent object properties, while the nodes are ontology concepts or instances of these. Each node and arc contains all the necessary information for the Evaluator/Integrator to execute sub-queries. That is: the XQuery (elaborated from the mapping) corresponding to the sub-query of the node or the arc, the names and the URLs of the Data Service of interest. An example is shown in (Figure 9).

The *YeastMed *Data Services are executed by the Evaluator/Integrator following the plan, after optimisation, generated by the Planner. In our case, SGD Data Service receives the first sub-query, because the object property *hasDescription *is mapped to the SGD Schema. TOP3 is returned as an answer of this sub-query and then is used by the sub-query *RegulatedBy *to find instances of *TranscriptionFactor*. The Yeastract Data Service is invoked this time because the property is mapped to the Yeastract Schema. Three instances of the type *TranscriptionFactor *are returned: Fhl1p, Hsf1p and Swi4p. For each of these instances, the Yeastract Data Service is called again. It receives this time the sub-query represented by the property *belongsTo *that contains the two arguments instantiated: the first one is one of the three instances returned by the previous query, and the second argument is instantiated by the name of the chromosome 16. This sub-query checks whether the Transcription factor has its coding gene on the chromosome 16. Only the instance Fhl1p is maintained. Finally the sub-query *hasPhosphoSite *is executed on the PhosphoGrid Data Service that returns all the *PhosphoSite *instances of the Transcription Factor Fhl1p. At each execution, the Evaluator/Integrator receives results in XML format from the target Data Services.

These results are instances of the XML schemas of the underlying sources. Based on the mapping between the elements of the source schemas and the elements of the ontology, these XML schema instances are translated into ontology instances which are not interconnected because they have been produced by different Data Services. To associate them, the Evaluator/Integrator uses just the instances of the domain and range classes of the object properties. The final result is an ontology instance that includes all the data extracted from the interrogated data sources. That is all the instances of the concepts *BibRef *of the protein TOP3 and all the *PhosphoSite *objects of the Transcription Factor Fhl1p.

## Results

We have conducted a usability assessment in order to grade how well biologists can learn and use YeastMed to achieve their goals and how satisfied they are with the system. We have also conducted a performance study of the system to reveal how run times behave towards the increase of the number of implied data sources in queries. In this section we present results obtained from these two studies.

### System usability

A variety of methods have been reported in the literature for assessing the perceived usability of interactive systems. We can particularly cite QUIS [45], SUS [46], CSUQ [47] and Microsoft's Product Reaction Cards [48]. Tullis and Stetson [49] reported a study that compared these methods and showed that the accuracy of the analysis increases as the number of participants gets larger (for a sample of 6 to 14) and that the accuracy of SUS increases quicker than the others. For that, we have used SUS method in our study. The SUS questionnaire consists of 10 items to which participants rate their level of agreement. Odd-numbered items are positively worded and even-numbered items are negatively worded. A 5-point scale of agreements numbered from 1 (anchored with "Strongly disagree") to 5 (anchored with "Strongly agree") is used for each. Each item's score contribution will range from 0 to 4. For odd-numbered items, the score contribution is the scale position minus 1. For even-numbered items, the score contribution is 5 minus the scale position. To get the overall SUS score, which is the indicator of usability, the sum of the item score contributions is multiplied by 2.5. SUS scores ranges from 0 to 100, with 100 representing a perfect score.

The usability study we conducted had two objectives: (1) having a general indicator on the usability of *YeastMed*, and (2) assessing the evolution of the system usability with the level of familiarisation to biological databases. This is represented, in our study, by the frequency of using biological databases of the participants. These objectives will let us (1) to grade how well biologists, in general, can learn and use the system and (2) how well we have succeeded to furnish an easy-to-use system for biologists who are familiar with HTML forms of biological databases.

**Figure 10:**
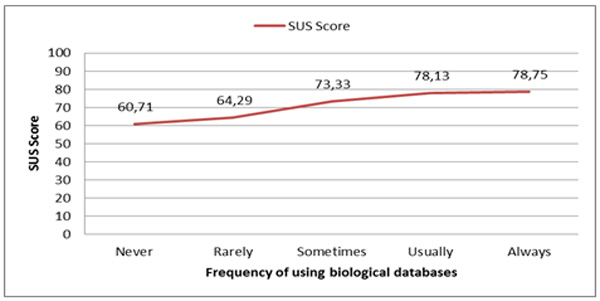
**SUS scores of YeastMed in function of the frequencies of using biological database**. The graph shows that the evolution of the mean SUS scores increases progressively when the frequency of using biological databases of biologists increases.

There were a total of 39 participants. Each one tested *YeastMed *before completing the SUS questionnaire. All the participants are biologists spread over 5 groups with different levels of familiarisation to biological databases. These groups contained between 7 and 9 participants and are named following the participants frequency of using biological databases, i.e. Never, Rarely, Sometimes, Usually and Always. For each participant we have calculated the individual SUS score and then the mean score for each group was determined. As shown in (Figure 10), the usability of *YeastMed *increases with the familiarisation to biological databases: The mean SUS score passes from 60.71 for biologists who never used biological databases to 78.75 for biologists who are always using biological databases with an overall SUS score of 71.54. With these scores, we can say that *YeastMed*, with its simple HTML form-based interface, is a system easy-to-use for biologists who are familiar to biological databases interfaces with a relatively lower usability for biologists with lower familiarisation.

### System performance

**Figure 11:**
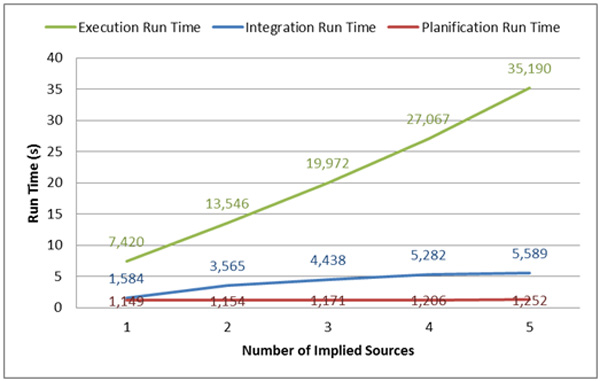
**Run times performed by YeastMed to answer a query**. The figure shows how run times of the three main stages required to answer queries behave when the number of the implied sources increases.

To illustrate the performance of *YeastMed*, we present, in this section, the result of a study conducted on the run times of the three main stages of the *YeastMed *query processing: Planification, Execution and Integration. The study concerned 50 queries distributed on 5 groups following the number of data sources participating in the query answer (from 1 to 5). All queries were run on a dual-processor 2.33 GHz Pentium 4 processor machine with 4 GB of memory. The objective of this study is not to provide a thorough performance analysis, but simply to show how run times behave towards the increase of the number of implied data sources. Each query was executed in three instances before to calculate its mean run times for the three stages. The data sources implied in the multi-sources queries are called exactly one time for each query. This had the objective to give certain uniformities to the study.

(Figure 11) illustrates the obtained results. It shows that there are no big changes in the Planification times when the number of the implied sources increases. The Planification time passes from 1.149 seconds for queries implying one source to 1.252 seconds for others that call 5 sources. In contrast, the execution time behaves differently. It increases with the number of the implied sources. This was expected because the execution of sub-queries in *YeastMed *makes use of a set of web services which are not called simultaneously but serially due to the fact that the call of a web service might depend on the result of another. As to the Integration run time, it shows also some increases but small compared to the Execution run time. It passes from 1.149 seconds for one-source-based queries to 5.589 seconds for queries implying 5 data sources. In *YeastMed*, the Integration stage is solicited even if just one source is implied. This is because, in addition to the integration process, it performs the transformation of the XML result returned by the web services to an RDF instance of the *YeastMed *ontology.

## Discussion

Dynamic integration is a very important issue for traditional mediator-based systems. They are usually developed as monolithic systems and their architecture based on wrappers involves a high degree of coupling among the system components. They usually do not provide scalable and reusable solutions. By the modular design and the uncoupling of all the components of *YeastMed*, we have sought to break out of traditional mediation architecture and provide a flexible platform for integrating Yeast data sources. The modular structure of the system reduces the costs of the system maintenance. The system can be easily extended to cover other sources. It is not required to rebuild the system from scratch. The new source components are built independently and then integrated in the system, i.e. publishing a web service underlying that source, adding semantic views on the source to the ontology, and publishing its mapping rules in the Semantic Directory. The rest of the system components are not touched. On the other hand, the fact that the system adopts a mediation approach avoids the updating problem when a change is made in a source at the level of data, because the system does not have a local copy of data. But when the change touches the structure of the flat files or the HTML pages from which *YeastMed *extracts data, the system will need to reflect this on its components, but only on the modified-source components: the modified-source schema, the mapping rules implying that source, the source-related entities in the ontology and also the web service of the source. The other source components are not modified and the system is not rebuilt from scratch.

Relying on Data Services rather than classical wrappers offers the possibility of reusing them by other mediators or any other data accessing application. This is the case of the quick search service we are proposing in the web site of *YeastMed*. This service is an added value of the system architecture. It makes use independently of the Data Services to look for entries and direct access to the integrated data sources without passing through the system mediator.

While some mediator-based systems require a specific query language or propose a complicated graphical user interface [25,27,29], the *YeastMed *mediator receives conjunctive queries expressed in terms of the ontology. Even though we estimate that these are not very complicated for biologists to express their requests, we have proposed a simple interface where requests are expressed using natural language through simple forms. All the required translations to conjunctive queries are hidden from users.

It is known that biologists have their own preferences toward databases [50]. In *YeastMed *we take this into account by giving users the possibility to specify from which database they prefer to get answers. The *YeastMed *ontology includes some source-related concepts which permit the user to express preferences on data sources. For example, a user can specify SGD as a source from which to get entries by selecting *SGDEntry *in the query form. Specifying a data source does not mean reducing the constraints to be only applied on the data of that source. Users can specify a source from which to get entries and apply constraints on related data from other sources. For example a user can ask for entries from *SGD *describing a chromosomal feature regulated by a transcription factor having the standard name *Rtg1*. This is translated into the following conjunctive query:

Ans(E) := SGDEntry(E),describes(E,F), ChromosomalFeature(F), RegulatedBy(F,R),TranscriptionFactor(R),hasName(R, "Rtg1");

In this conjunctive query, SGD entries are solicited, but all the constraints are made on data residing in Yeastract (data related to Transcription Factor). If the SGD entry has not been specified, the result entries will be returned by default from Yeastract. *YeastMed *is able to find the equivalent of such entries (if any) in SGD.

## Conclusions

We have described *YeastMed*: an XML and mediator-based system that Integrates five Yeast databases which have the most appropriate properties for studying *Saccharomyces cerevisiae*.

Data Services play an important role in the integration process of this system, where they are considered as an interface which receives queries, accesses to a data source, extracts data and translates them into a common data model used by SB-KOM. In *YeastMed*, Data Services extract data mainly from flat files because most of the integrated data sources are accessible via ftp mechanisms and provide data in tabular or XML format. This reduces the costs of the maintainability of the system because flat files structures are not frequently target to changes.

In our system, the schema integrator is an ontology and the results are ontology instances. The use of the ontology and instances enables basic reasoning processes (class-subclass inference) to be later included. This will permit *YeastMed *to infer new relationships between the instances of the ontology when solving a user query and thus, discover new knowledge for the query answers. The final result is an ontology instance that includes all the data extracted from the integrated data sources. It is converted to an HTML Format before to be presented to users.

The objectives expected from the *YeastMed *system are not yet all met. The system is still in its natal phase and additional work is undertaken to improve it. The system does not yet make all its ontology available to users when formulating queries. This is because it is not yet able to answer queries expressed in terms of some part of the ontology. In addition, the fact that the system answers only conjunctive queries limits the user requests expression; i.e. it is not able to answer queries using disjunction quantification (or union operator which is denoted as ∪).

## List of abbreviations

API: Application Programming Interface; CDS: Coding Sequence; CPL: Collection Programming Language; CSUQ: Computer System Usability Questionnaire; CYGD: the Comprehensive Yeast Genome Database; DTD: Document Type Definition; ETL: Extract, Transform, Load; FTP: File Transfer Protocol; GAV: Global as view; GO: Gene ontology; GUS: Genomics Unified Schema; HTML: Hypertext Markup Language; LAV: Local as view; MIPS: Munich Information center for Protein Sequences; OWL: Web Ontology Language; QIF: Query Internal Form; QUIS: Questionnaire For User Interaction Satisfaction; RDF: Resource Description Framework; RDFS: Resource Description Framework Schema; RNA: Ribonucleic Acid; SB-KOM: System Biology Khaos Ontology-based Mediator; SGD: Saccharomyces Genome Database; SQL: Structured Query Language; SUS: System Usability Scale; TAMBIS: Transparent Access to Multiple Bioinformatics Information Sources; XQuery: XML Query Language; XML: Extensible Markup Language; XMLDSS: XML DataSource Schema: WSDL: Web Service Description Language.

## Competing interests

The authors declare that they have no competing interests.

## Authors' contributions

JFAM, BDRH and KL carried out the initial purpose of using mediation-approach to integrate yeast data, followed and tested the work. AB and KM performed the technical design, implemented and tested the system and drafted the manuscript. IND performed the design task and helped to draft the manuscript. AK ensured technical supervision and support. All authors read and approved the final manuscript.
